# A potential panel of two-long non-coding RNA signature to predict recurrence of patients with laryngeal cancer

**DOI:** 10.18632/oncotarget.18751

**Published:** 2017-06-28

**Authors:** Zhigang Bai, Enhong Shi, Qiwei Wang, Zhouwei Dong, Ping Xu

**Affiliations:** ^1^ Department of Otorhinolaryngology, Head and Neck Surgery, The Fourth Affiliated Hospital of Harbin Medical University, Harbin 150001, China; ^2^ Department of Medical Oncology, Heilongjiang Province Hospital, Harbin 150001, China; ^3^ Department of Otorhinolaryngology, Head and Neck Surgery, The First Affiliated Hospital of Harbin Medical University, Harbin 150001, China; ^4^ Department of Otorhinolaryngology, Head and Neck Surgery, The Fourth Hospital of Harbin, Harbin 150070, China

**Keywords:** disease recurrence, laryngeal cancer, long non-coding RNAs, survival

## Abstract

Accumulating evidence has shown that aberrant lncRNA expression plays an oncogenic or tumor-suppressive role in the tumorigenesis of laryngeal cancer. However, the prognostic roles of lncRNAs in laryngeal cancer recurrence are still poorly understood. In this study, we obtained lncRNA expression profiles of 109 patients with laryngeal cancer by mining previously published gene expression microarray data from the Gene Expression Omnibus (GEO) and identified two lncRNAs associated with laryngeal cancer recurrence in the training dataset by using Cox regression analysis. Then these two lncRNAs were combined to a two-lncRNA signature for identifying patients at high-risk of disease recurrence. By applying this two-lncRNA signature to the testing dataset, a clear separation was observed in the survival curves between patients with low- or high-risk scores, indicating good reproducibility of this two-lncRNA signature in predicting disease-free survival of laryngeal cancer. Further analysis revealed that the prognostic value of the two-lncRNA signature was independent of other clinical features, including age, stage and grade. Subsequent gene set enrichment analysis suggested that the two-lncRNA signature was more likely to involve with GPCRs downstream signaling pathway, potassium channel pathway and aurora-A pathway. Our study demonstrated that the two-lncRNA signature may be a novel potential biomarker for prognosis of laryngeal cancer and may provide novel insights into the molecular mechanism of laryngeal cancer.

## INTRODUCTION

Head and neck cancer is one of the commonly diagnosed cancers all over the world. Laryngeal cancer is one of the most common types of head and neck cancer and has 4-fold higher incidence rates in men compared with women, accounting for about 0.79% of estimated numbers of newly diagnosed cases of invasive cancer expected in the United States in 2017 [1]. Surgery treatment combining radiotherapy and chemotherapy is the current treatment options and has been shown to improve the prognosis and survival of patients with Laryngeal cancer. However, recurrent laryngeal carcinoma is a common clinical problem faced by the physicians [2]. Therefore, assessing the recurrence risk of laryngeal cancer for each patient would greatly accelerate progress toward early detection of recurrent disease and lead to targeted treatment options and better survival rates for patients.

Long non-coding RNAs (lncRNAs), a novel class of non-coding RNAs, are defined arbitrarily as non-coding transcripts longer than 200 nucleotides [3]. There is increasing evidence that lncRNAs have crucial roles in transcriptional regulation and epigenetic gene regulation during both developmental and differentiation processes [4]. More and more high-throughput profiles or RNA sequencing experiments have identified a large number of differentially expressed lncRNAs in cancers which are becoming recognized as a hallmark feature of cancer [5, 6]. Some of the aberrantly expressed lncRNAs have been found to be oncogenes or tumor suppressors to influence cancer phenotypes, such as GAS5, PCA3, MALAT1, H19, PANDAR and so on [7]. Like mRNA and microRNA, lncRNAs have demonstrated utility as molecular markers in cancer diagnostics and prognostics, thus provide a potential avenue and resource in terms of developing novel biomarkers [8–24]. Previous microarray-based study of 87 laryngeal squamous cell carcinoma samples and paired adjacent normal tissue have identified hundreds of differentially expressed lncRNAs which highlighted the clinical significance of lncRNAs in laryngeal cancer [25]. Another study by Wu *et al.* found that *H19* were inversely correlated with the survival rate of patients with laryngeal squamous cell carcinoma [26].

To further investigate the predictive value of lncRNAs for disease recurrence in laryngeal cancer, we mined previously published gene expression microarray data from the Gene Expression Omnibus (GEO), and conducted lncRNA profiling on a cohort of 109 patients with laryngeal cancer. By using the sample-splitting method and Cox regression analysis, we identified a two-lncRNA signature associated with disease-free survival, and then established a risk score formula using the expressions of these two lncRNAs in the training dataset. The prognostic value of the two-lncRNA signature was further confirmed in the testing dataset.

## RESULTS

### Identification of potential prognostic lncRNAs associated with laryngeal cancer recurrence in the training dataset

**Figure 1:**
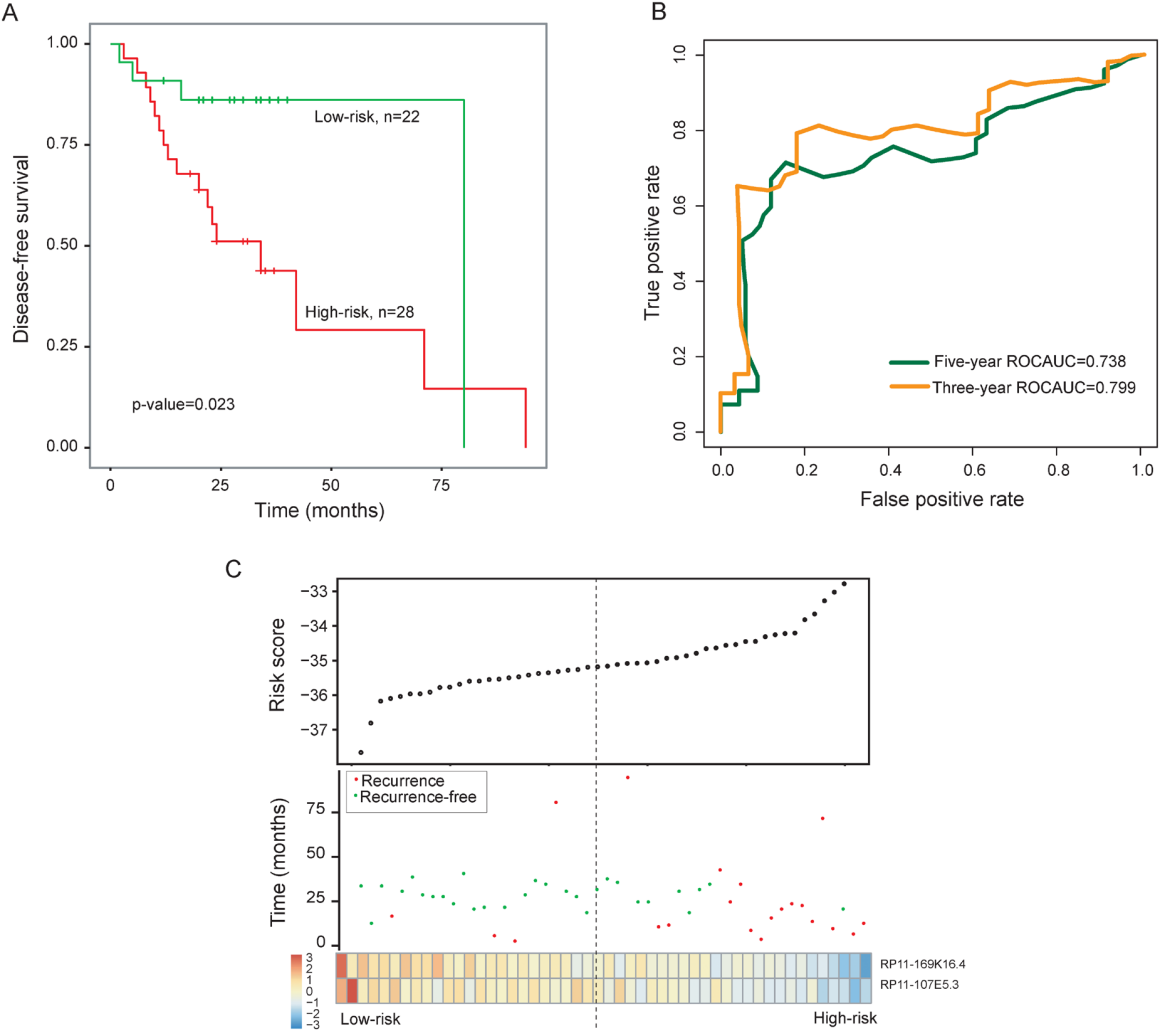
Prognostic assessment of the two-lncRNA signature in the training dataset **(A)** Kaplan-Meier survival curves of overall survival between high-risk group and low-risk group in the training dataset. **(B)** Time-dependent ROC curves of the two-lncRNA signature at 36 and 60 months of disease-free survival. **(C)** Presentation of risk scores, survival status and lncRNA expression pattern in high-risk and low-risk groups

In order to identify potential prognostic lncRNAs associated with laryngeal cancer recurrence, we performed univariate Cox regression analysis to investigate the association between lncRNA expression and DFS in the training dataset. A total of seven lncRNAs were found to be significantly associated with disease-free survival in the training dataset (p<0.01) (Table 1). As shown in Figure 1, the negative coefficients of the univariate analysis indicated that high expression levels of these seven lncRNAs were associated with longer disease-free survival, suggesting their tumor suppressor role in laryngeal cancer recurrence. In order to evaluate whether these even lncRNAs have independent prognostic value to predict patients’ disease-free survival when considering the mutual effect among them, we subjected these seven lncRNAs into the multivariate Cox regression analysis with DFS as a dependent variable. Finally, only two of seven lncRNAs (*RP11-169K16.4* and *RP11-107E5.3*) showed predictive power and could independently predict patients’ DFS at a statistically significant level of 0.1 (Figure 1).

**Table 1:** Seven lncRNAs significantly associated with disease-free survival in the training dataset

Ensemble ID	Gene name	Chromosome	P-value	HR	coefficient
ENSG00000224459.1	RP11-169K16.4	Chr 1: 15,740,051-15,749,896(-)	0.001	0.03	-3.577
ENSG00000248525.2	CTD-2001E22.1	Chr 5: 9,621,377-9,658,458(-)	0.004	0.04	-3.275
ENSG00000227907.1	RP11-102C16.3	Chr 1: 167,052,551-167,058,542(+)	0.005	0.07	-2.726
ENSG00000279166.1	RP11-107E5.3	Chr 2: 144,494,265-144,496,878(-)	0.007	0.04	-12.538
ENSG00000254488.1	RP11-65G9.1	Chr Y: 21,038,289-21,044,724(-)	0.008	0.15	-1.909
ENSG00000280211.1	RP11-2C24.3	Chr 16: 30,773,532-30,776,033(-)	0.009	0.01	-4.912
ENSG00000249717.1	RP11-44F21.3	Chr 4: 74,955,974-74,970,362 (-)	0.010	0.07	-2.694

### Derivation of a two-lncRNA prognostic signature from the training dataset as a potential indicator for predicting recurrence

To construct a lncRNA signature for clinical applicability, these two prognostic lncRNAs were fitted in a multivariable Cox regression model in the training dataset to obtain their relative contribution for predicting recurrence of LC. A two-lncRNA signature was created by including each of two prognostic lncRNAs, weighted by their estimated regression coefficients in the above multivariable Cox regression analysis as follows: two-lncRNA signature= (-2.6722 * RP11-169K16.4)+(-7.4706 * RP11-107E5.3). We calculated a risk score for each patient of the training dataset using the two-lncRNA signature. Using the median risk score as the cutoff, patients were classified into a high-risk group and a low-risk group. The patients with high-risk scores were expected to be at high-risk of LC recurrence. As a result, patients with the high-risk signature had significantly shorter disease-free survival than those with the low-risk signature (median survival 34 months vs. 80 months; p=0.023, log-rank test) (Figure 1A). Time-dependent ROC curves were used to assess the prognostic power of the two-lncRNA signature in predicting recurrence. The AUC for the two-lncRNA signature prognostic model was 0.799 and 0.738 at 36 and 60 months of disease-free survival (Figure 1B). The disease-free survival rates at three- and five-years in the high-risk group is 43.8% and 29.2%, respectively, whereas the corresponding survival rates in the low-risk group were both 90.9%, respectively. The association of the two-lncRNA risk score with disease-free survival was also significant when it was evaluated as a continuous variable in the univariate Cox regression analysis (p-value <0.001, HR = 2.72, 95% CI = 1.60-4.62).

The distribution of two-lncRNA signature risk scores, recurrence status and expression pattern was shown in Figure 1C. As shown in Figure 1C, we found that these two lncRNAs were expressed with low levels in patients with high-risk scores, whereas these two lncRNAs were expressed with high levels in patients with low-risk scores.

### Validation of the two-lncRNA signature for recurrence prediction in the testing dataset and entire GSE27020 dataset

**Figure 2:**
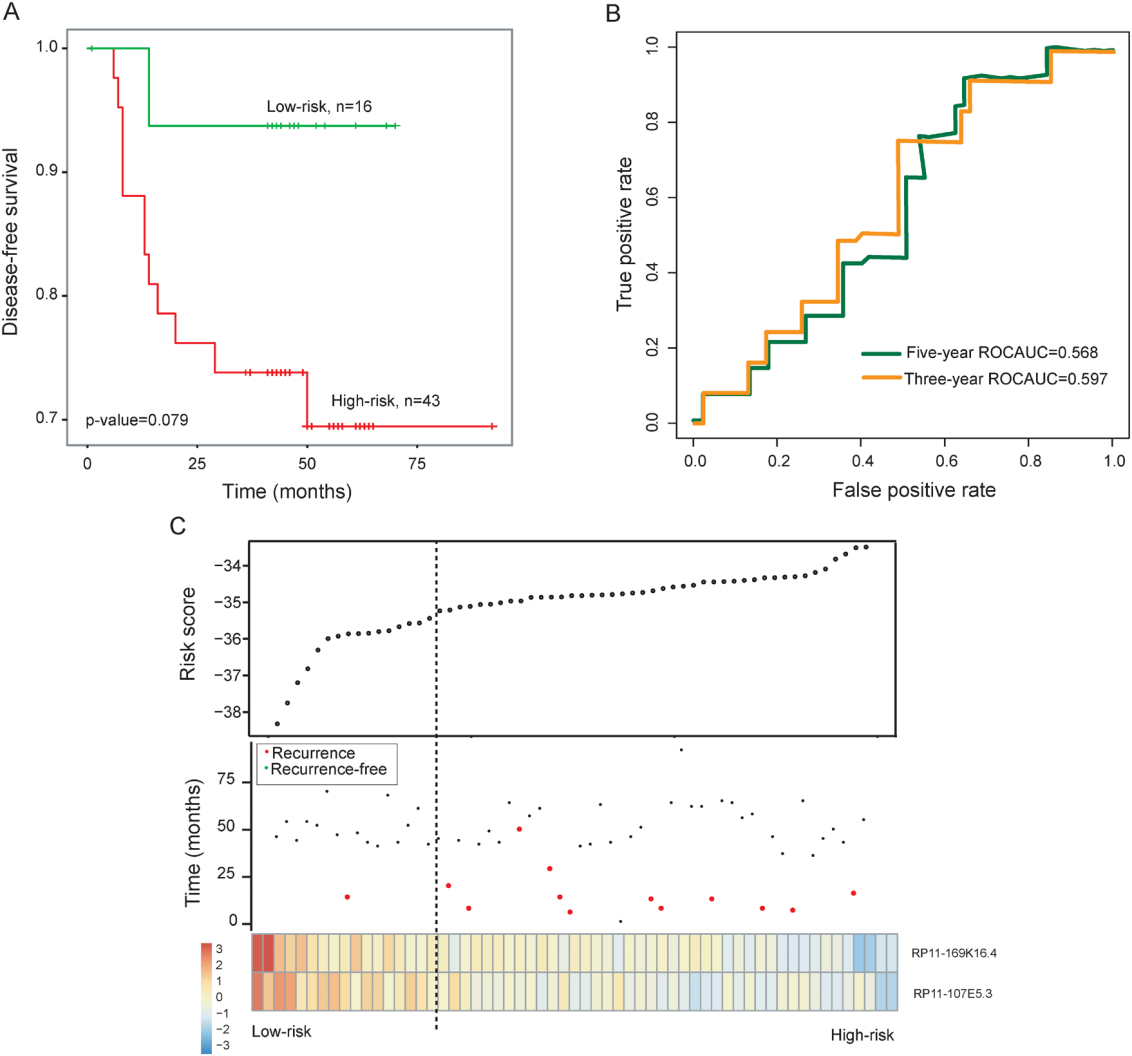
Prognostic assessment of the two-lncRNA signature in the testing dataset **(A)** Kaplan-Meier survival curves of overall survival between high-risk group and low-risk group in the testing dataset. **(B)** Time-dependent ROC curves of the two-lncRNA signature at 36 and 60 months of disease-free survival. **(C)** Presentation of risk scores, survival status and lncRNA expression pattern in high-risk and low-risk groups

To further test the prognostic power of the two-lncRNA signature in recurrence prediction, the two-lncRNA signature was validated in the independent testing dataset. By using the same two-lncRNA signature risk score formula derived from the training dataset, each of patients in the testing dataset was assigned a risk score and was classified into a high-risk group (n=43) or a low-risk group (n=16) using the same cutoff point as for the training dataset. Consistent with our findings in the training dataset, the disease-free survival of the high-risk group patients was marginally significantly shorter than that of low-risk group patients (p=0.079, log-rank test) (Figure 2A). The disease-free survival rates at three- and five-years in the high-risk group is 73.8% and 69.5%, respectively, whereas the corresponding survival rates in the low-risk group were both 93.75%, respectively. The AUC for the two-lncRNA signature prognostic model was 0.597 and 0.568 at 36 and 60 months of disease-free survival (Figure 2B).

**Figure 3:**
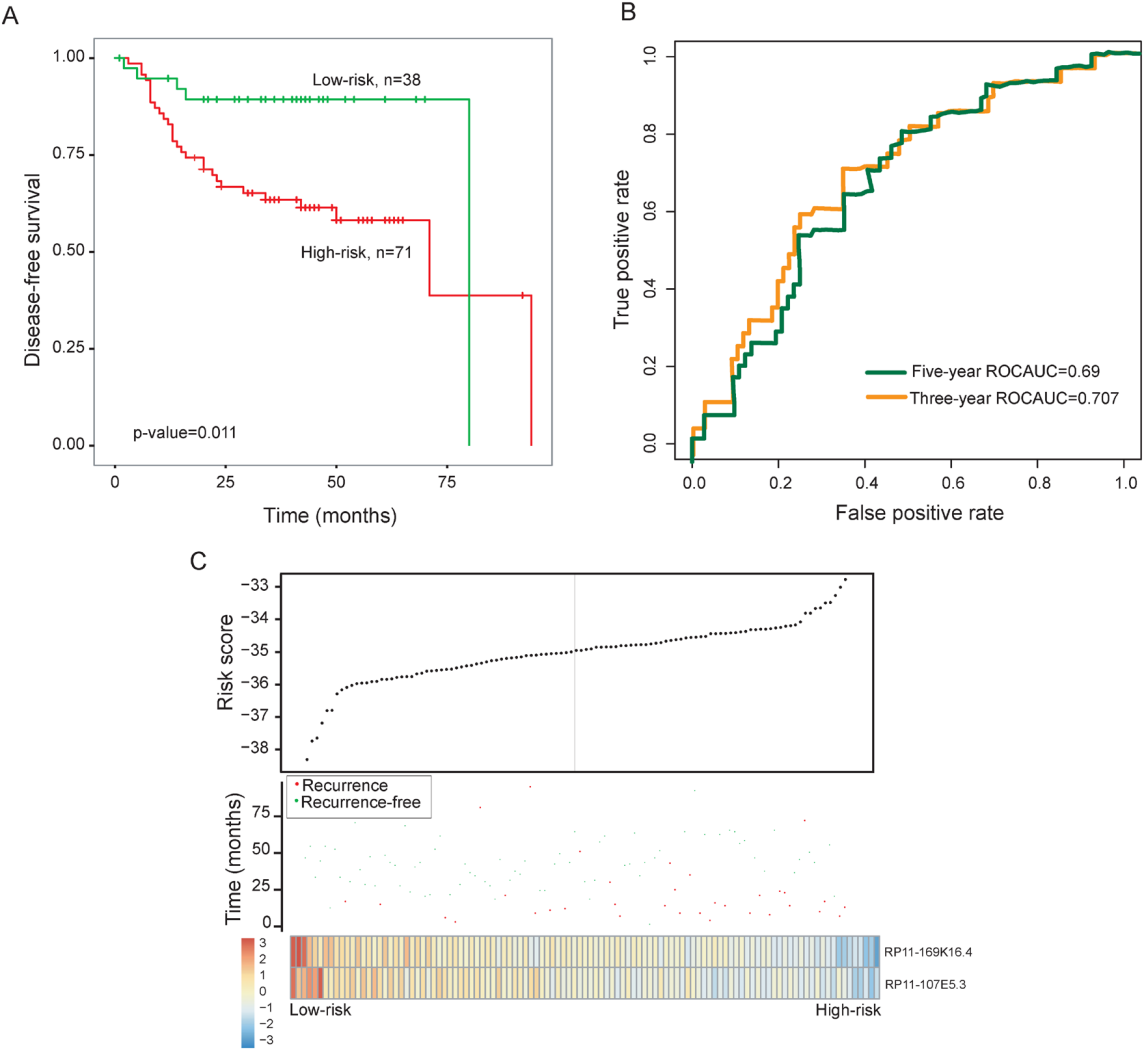
Prognostic assessment of the two-lncRNA signature in the entire GSE27020 dataset **(A)** Kaplan-Meier survival curves of overall survival between high-risk group and low-risk group in the entire GSE27020 dataset. **(B)** Time-dependent ROC curves of the two-lncRNA signature at 36 and 60 months of disease-free survival. **(C)** Presentation of risk scores, survival status and lncRNA expression pattern in high-risk and low-risk groups

The two-lncRNA signature was then tested for its predictive value in the entire GSE27020 dataset of 109 patients with laryngeal cancer. Risk score-based classification of the entire GSE27020 dataset (i.e. combined training and testing datasets) also yielded similar results. Patients with the high-risk scores had significantly shorter disease-free survival than those with the low-risk scores (median survival 71 months vs. 80 months, p=0.011, log-rank test) (Figure 3A). The disease-free survival rates at three- and five-years in the high-risk group is 63.5% and 58.2%, respectively, whereas the corresponding survival rates in the low-risk group were both 89.3%, respectively. The AUC for the two-lncRNA signature prognostic model was 0.707 and 0.69 at 36 and 60 months of disease-free survival (Figure 3B).

The distribution of two-lncRNA signature risk scores, recurrence status and expression pattern were analyzed in the testing dataset and entire GSE27020 dataset separately, and the results were similar to those observed in the training dataset above (Figure 2C and 3C).

### **Independence** of prognostic value of two-lncRNA signature from other clinical features

We further investigated whether the prognostic value of two-lncRNA signature was independent of other clinical features. The two-lncRNA signature risk score, age, stage and grade were defined as covariates. The effect of the two-lncRNA signature risk score, age, stage and grade on disease-free survival was evaluated using multivariate Cox regression analysis. The results showed that only the two-lncRNA signature risk score is an independent predictor of patient’s disease-free survival (p-value = 0.002, HR = 2.21, 95% CI = 1.40-3.49) (Table 2). The results of the multivariable Cox regression analysis thus indicated that the predictive ability of the two-lncRNA signature is independent of other clinical features for the DFS of patients with laryngeal cancer.

**Table 2:** Univariate and multivariate Cox regression analysis

Variables	Univariate analysis			Multivariate analysis		
	HR	95% CI	p-value	HR	95% CI	p-value
**Risk score**	2.21	1.40-3.49	<0.001	2.12	1.33-3.37	0.002
Age	1.020	0.99-1.06	0.19	1.01	0.98-1.05	0.46
Stage						
III/IV	1 (reference)			1 (reference)		
I/II	0.96	0.45-2.06	0.92	0.98	0.45-2.16	0.97
Grade						
G3	1 (reference)			1 (reference)		
G1/G2	0.88	0.34-2.30	0.8	0.99	0.37-2.64	0.98

### Functional implication of two-lncRNA signature

**Figure 4:**
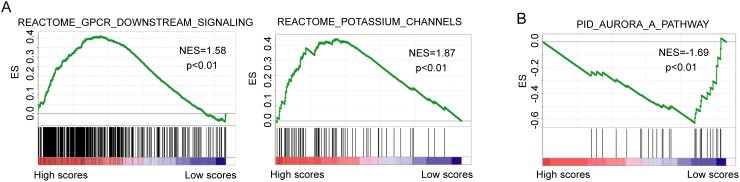
Gene set enrichment analysis from gene expression profiles of 109 patients in the high-risk and low-risk groups **(A)**Enriched biological pathways associated with high-risk scores. **(B)** Enriched biological pathways associated with low-risk scores.

A gene set enrichment analysis (GSEA) [27] was performed on from gene expression profiles of 109 patients in the high-risk and low-risk groups classified by the two-lncRNA signature to identify the potential biological pathways associated with the two-lncRNA signature (p<0.01). The high-risk scores were associated with coordinated transcriptional up-regulation of GPCRs downstream signaling pathway and potassium channel pathway (Figure 4A). The low-risk score was accompanied by up-regulation of Aurora-A pathway (Figure 4B), all of which involved in human cancers.

## DISCUSSION

Laryngeal cancer is the eleventh most common type of cancer in men worldwide. Patients treated by the primary therapy are at the highest risk of recurrence in the first two to three years and local recurrence is the major manifestation of treatment failure in patients with laryngeal cancer. Adjuvant therapy (such as radiotherapy and chemotherapy) after surgery treatment has been used to decrease the chance of disease recurrence [28]. However, the risk of recurrence varies considerably in laryngeal cancer patients. For example, the rate of recurrence for laryngeal cancer patients with early stage ranges from 5%-30% advanced-stage laryngeal cancer patients is approximately 30%-50% [29, 30]. Therefore, some of the laryngeal cancer patients with good prognosis without adjuvant therapy suffer from side effects of laryngeal cancer that affects the quality of life. There is a need for specific prediction of recurrence risk to ensure that patients will receive more personalized and appropriate treatment. However, established clinicopathological factors can not sufficiently predict patients that will recur. To data, many research efforts have been made to identify mRNA or miRNA signature in the risk stratification of laryngeal cancer with unfavorable prognosis. For example, Fountzilas *et al.* analyzed the gene expression profiles of 66 patients with laryngeal cancer treated locally with surgery and developed a 28-gene prognostic model to predict the risk of disease recurrence in primary laryngeal cancer [31]. Another two-miRNA (hsa-miR-657 and hsa-miR-1287) signature were identified by microarray analysis to act as an effective predictive molecular biomarker for early diagnosis of laryngeal cancer [32].

lncRNAs, a novel class of ncRNAs, is becoming recognized as ideal candidates for the development of novel biomarkers in diagnostics and prognostics in various human cancers. Expression analysis of lncRNAs demonstrated their more restricted tissue-specific and cancer-specific expression patterns than the expression of protein-coding genes and miRNAs, indicating their superiority as potential biomarkers predictive of prognosis or response to therapy [11]. Recent studies have found that aberrant lncRNA expression plays an oncogenic or tumor-suppressive role in the tumorigenesis of laryngeal cancer and may serve as potential biomarkers for laryngeal cancer [25, 26, 33]. However, the prognostic roles of lncRNAs in laryngeal cancer recurrence are still poorly understood. Therefore, in this study we obtained lncRNA expression profiles of 109 patients with laryngeal cancer by mining previously published gene expression microarray data from the Gene Expression Omnibus (GEO) and identified two lncRNAs associated with laryngeal cancer recurrence in the training dataset by using Cox regression analysis. Based on our knowledge, this is the first report that relates lncRNA expression pattern with the recurrence of laryngeal cancer. Then these two lncRNAs were combined to a two-lncRNA signature for identifying patients at high-risk of disease recurrence. By applying this two-lncRNA signature to the testing dataset, a clear separation was observed in the survival curves between patients with low- or high-risk scores. Patients with low-risk scores tended to have prolonged disease-free survival, whereas patients with high-risk scores tended to have shortened disease-free survival, indicating good reproducibility of this two-lncRNA signature in predicting disease-free survival of laryngeal cancer. Further analysis revealed that the prognostic value of the two-lncRNA signature was independent of other clinical features, including age, stage and grade.

Through the gene set enrichment analysis from gene expression profiles of 109 patients in the high-risk and low-risk groups classified by the two-lncRNA signature, we found that the two-lncRNA signature was more likely to involve with GPCRs downstream signaling pathway, potassium channel pathway and aurora-A pathway. Previous studies have revealed a crucial role of GPCRs and their downstream signaling targets in multiple physiological functions as well as in tumor growth and metastasis [34]. Potassium channels have emerged as regulators of both cell cycle and cell proliferation [35]. The Kv3.4 potassium channel subunit has been observed to be frequently increased during head and neck squamous cell carcinomas tumourigenesis and correlated significantly with a higher cancer risk [36]. Aurora-A, also designated as STK15/STK6, is a serine/threonine kinase that plays a crucial role in mitosis and spindle assembly during the various stage of mitosis. Aberrant amplification and/ or overexpression of Aurora-A has been reported in human malignancies. A recent study also found that suppression of Aurora-A-FLJ10540 signaling axis prohibits the malignant state of head and neck cancer [37].

The limitations should be acknowledged in this study. First, the two-lncRNA signature was identified and validated in the relatively small sample dataset. Therefore, the significance and robustness of the signature as a prognostic classification requires further confirmation independent larger cohorts in future studies. Second, coverage of lncRNA in this study is relatively low compared to known lncRNAs recorded in the GENCODE database. Third, further experimental studies should be conducted to investigate the functional roles of two-lncRNA signature.

In conclusion, our results showed that the two lncRNA signature significantly predicts disease-free survival of patients with laryngeal cancer in the GEO cohort, indicating that it may be a novel potential biomarker for prognosis of laryngeal cancer. Moreover, the two-lncRNA signature may provide novel insights into the molecular mechanism of laryngeal cancer.

## MATERIALS AND METHODS

### Patient datasets

The mRNA expression profiles of laryngeal cancer patients were retrieved from the publicly available GEO database (https://www.ncbi.nlm.nih.gov/geo/). After removing patients without disease-free survival and recurrence information, a total of 109 laryngeal cancer patients were obtained from the GSE27020 based on Affymetrix HG-U133A array (https://www.ncbi.nlm.nih.gov/geo/query/acc.cgi?acc=GSE27020) and their corresponding clinical were obtained from Fountzilas‘s study. These 109 laryngeal cancer patients were randomly divided into the training dataset composing of 50 patients and the testing dataset composing of 59 patients. The detailed clinical information of patients in the training and testing datasets were presented in Table 3.

**Table 3:** Clinicopathological characteristics of patients with laryngeal cancer

Characteristics	Training dataset (n=50)	Testing dataset (n=59)	Entire dataset (n=109)
**Age**	64.54±9.52	62.27±10.45	63.31±10.05
**Recurrence**			
Yes	29	46	75
No	21	13	34
**Stage**			
I	11	1	12
II	8	10	18
III	14	12	26
IV	17	26	43
**Grade**			
G1	23	19	42
G2	21	28	49
G3	6	10	16
**Radiation status**			
Yes	29	25	54
No	20	23	43
**Smoking status**			
Yes	50	58	108
No	0	1	1
**Gender**			
Male	49	55	104
Female	1	4	5

### LncRNA expression profile mining

The CEL file data of 109 LC were obtained from GSE27020 and processed using the Robust Multichip Average (RMA) method for background correction and quantile normalization. The raw probe intensities were log-2-scale transformed and standardized by transforming the expression data into having a mean of 0 and a standard deviation (SD) of 1. First, Affymetrix HG-U133A probesets from the Affymetrix website (http://www.affymetrix.com) were re-mapped to the human genome (GRCh38/hg38) using SeqMap33 with no mismatch [38]. Second, the chromosomal positions of those probes, which were uniquely mapped to the human genome, were matched to the chromosomal positions of lncRNAs derived from GENCODE (release 21, GRCh38) [23]. A total of 909 probes (or probe sets) and 649 corresponding lncRNA genes were obtained. Multiple probes (or probe sets) that mapped to the same lncRNA were combined using the median expression value of the probes (or probe sets).

### Statistical analysis

The univariate and multivariate Cox regression analysis were performed to evaluate the association between each of lncRNA and patient’s disease-free survival and identify independent lncRNAs significantly associated with DFS. Then a lncRNA signature was developed as a linear combination of the expression value of lncRNAs weighted by their respective multivariate Cox regression coefficients. According to the lncRNA signature, LC patients were classified into high-risk group with high-risk lncRNA signature and low-risk group with low-risk lncRNA signature using the median lncRNA signature risk score from the training dataset as the cutoff. Kaplan-Meier survival curve and log-rank test were used to compare the survival difference between the high-risk and low-risk groups in each dataset. Univariate and multivariate analyses with Cox proportional hazards regression for disease-free survival were performed on the individual clinical feature with and without the lncRNA signature in each dataset. Hazard ratios (HR) and 95% confidence intervals (CI) were calculated. Time-dependent ROC analysis was performed to compare the sensitivity and specificity of the recurrence prediction based on the lncRNA risk score. All analyses were performed using R/Bioconductor.

### Functional enrichment analysis

In order to explore the potential biological roles of lncRNA, Gene set enrichment analysis (GSEA) was performed by the JAVA program using MSigDB (c2.cp.v5.0, 1330 gene sets) to rank gene set associated with risk score by enrichment score [27]. The gene sets with positive enrichment score (or negative enrichment score) and p-value < 0.01 were considered as significantly enriched gene sets in which most of the genes are up-regulated accompanied with high-risk scores (or low-risk scores).
